# Preliminary Schools for Probationers

**Published:** 1909-03-06

**Authors:** 


### Nursing Administration.

### PRELIMINARY SCHOOLS FOR PROBATIONERS.

### 1.—NUMBERS.

To contrast the pupils passing out of their preliminary school into the wards with the raw probationers, who wear their uniforms for the first time, is to be struck with the overwhelming advantages which the school affords to the hospital. It was recently our privilege to inspect the group of pupils at Guy's Hospital at the moment when, having completed their preliminary course, they were passing from their first interview with the matron to commence their probation in the wards. Their bearing was composed and self-reliant, as befitted women who had learned the secret of discipline. They were passing on to take up work with which they were thoroughly conversant. Without having yet entered a ward they had become proficient in bandaging, the making of dressings, the use of instruments, bed-making, housework, sick-room cookery, and other elementary duties such as might be required from them in the first months of ward work. They had studied the elements of physiology, anatomy, and hygiene, and been exercised in the elementary duties of the dispensary, so far as the mixing of lotions, the measuring of doses, etc. They had all passed with credit an examination of which the following paper is a specimen:—

1. Give a full account of the structure of the heart, the composition of the blood, and the various functions of the blood.

2. Describe as accurately as you can the structure and blood-supply of the kidneys, and give an account of the way in which the urine is excreted, and enumerate the various constituents and characters of the urine.

3. Describe accurately those alimentary ferments which act upon proteid and their modification. Enumerate six different varieties of proteid food.

4. In as few words as possible indicate the meaning of the following: (1) The pleura. (2) Vesical (adjective). (3) Molar. (4) Emulsification. (5) Pulsation. (6) Peritoneum. (7) Ulna. (8) Axilla. (9) Popliteal space. (10) A digit. (11) Meninges. (12) Hepatic (adjective).

What is even more important, they had learned what they knew in an orderly manner, with entire concentration on the matter in hand, without worry, or distress of mind. Those who gave the instruction were at liberty to devote their whole attention to their pupils, without that horrible strain of divided duties which compels that the new probationer must perforce be left to her own devices rather than that the whole work of the ward be retarded while explanations are given, or patients suffer that novices be drilled. There can be no doubt that the good effect of these few orderly weeks of preliminary instruction are felt right through the nurse's career. It is true of the nurse's life as of most things that "Ce n'est que le premier pas qui coate." And "Well begun is half done."

Is the preliminary training school an impossible ideal only to be realised by hospitals with plenty of money to spare for such luxuries? There is no doubt that it can be managed very expensively, but it is the object of these articles to show that it can also be so organised that the whole cost is defrayed by the pupils, the school undoubtedly serving the additional purpose of attracting a good class of intending probationers.

In organising a preliminary school of this nature, the first thing to be done is to reckon out how many new probationers are required for the supply of the wards during the year. Even under the most advantageous circumstances probationers sometimes break their agreements on account of illness or for family reasons. And not all the pupils received will be found suitable to pass on to the wards. Hence accommodation must be provided for rather more pupils than under normal circumstances would fill normal vacancies. A course of six weeks will be found sufficient under skilled instruction to fit the pupil for commencing her duties as probationer, but the course may be extended to two months, or even to three if thought desirable. It must be remembered, however, that every additional week of instruction makes demands on the pupil's purse, and the number of opulent probationers is strictly limited. It is to the interest of the hospital to get a wide choice of candidates for the training school, and this is best secured by keeping the course as short as the exigencies of the teaching will admit. At Guy's Hospital the six weeks' course is found entirely satisfactory, and this has been selected as the basis also of the preliminary school recently founded at the Bristol Royal Infirmary. It is neither possible nor desirable to dovetail the relays of pupils one into another so that they succeed each other on successive days. Hence the seventh week of the course is conveniently allotted to the business of passing the candidates into the wards, and the school is renewed every seventh week. Under this arrangement seven relays of pupils can be received in the course of the year, and during one month in the summer the sister-in-charge takes her holiday and the school is empty. Supposing that 50 new probationers are required during the year, accommodation should be provided for eight pupils, that 56 may be available. As the year goes on, should it appear that vacancies will not occur for all the pupils at this rate, the number could be brought down to seven, but it is better to have vacancies for training one or two more than are wanted than to be forced to introduce untrained women into the wards. If the course is extended to eight weeks, only six relays can be trained in the year, and increased accommodation will be needed. When the numbers to be trained simultaneously sink below seven or eight, it hardly pays to provide a sister devoting the whole of her time to this work. Under such circumstances, we believe it might be found possible to keep the school open for part of the year only, the sister-in-charge being employed at other times in taking holiday or other duty. A hospital requiring only 20 or 25 probationers might arrange to train three relays, and even those needing but eight or ten probationers might manage to arrange a school term for them before passing them on into the wards.

